# Weight Change and Mortality Risk in Heart Failure With Preserved Ejection Fraction

**DOI:** 10.3389/fcvm.2021.681726

**Published:** 2021-06-04

**Authors:** Peisen Huang, Zejun Guo, Weihao Liang, Yuzhong Wu, Jingjing Zhao, Xin He, Wengen Zhu, Chen Liu, Yugang Dong, Yuan Yu, Bin Dong

**Affiliations:** ^1^Department of Cardiology, The First Affiliated Hospital of Sun Yat-sen University, Guangzhou, China; ^2^National Health Commission Key Laboratory of Assisted Circulation (Sun Yat-sen University), Guangzhou, China; ^3^National-Guangdong Joint Engineering Laboratory for Diagnosis and Treatment of Vascular Diseases, Guangzhou, China; ^4^The Hospital of South China Normal University, Guangzhou, China; ^5^Guangdong Provincial People's Hospital, Guangdong Academy of Medical Sciences, Guangzhou, China; ^6^Department of Cardiology, Guangdong Cardiovascular Institute, Guangzhou, China

**Keywords:** HFpEF, weight gain, weight loss, mortality, heart failure

## Abstract

**Aims:** The aim of the study was to determine the associations of weight loss or gain with all-cause mortality risk in heart failure with preserved ejection fraction (HFpEF).

**Methods and Results:** Non-lean patients from the Americas from the Treatment of Preserved Cardiac Function Heart Failure with an Aldosterone Antagonist study were analyzed (*n* = 1,515). Weight loss and weight gain were defined as a decrease or increase in weight ≥5% between baseline and 1 year. To determine the associations of weight change and mortality risk, we used adjusted Cox proportional hazards models and restricted cubic spline models. The mean age was 71.5 (9.6) years. Weight loss and gain were witnessed in 19.3 and 15.9% patients, respectively. After multivariable adjustment, weight loss was associated with higher risk of mortality (HR 1.42, 95% CI 1.06–1.89, *P* = 0.002); weight gain had similar risk of mortality (HR 0.98, 95% CI 0.68–1.42, *P* = 0.932) compared with weight stability. There was linear relationship between weight change and mortality risk. The association of weight loss and mortality was different for patients with and without diabetes mellitus (interaction *p* = 0.009).

**Conclusion:** Among patients with HFpEF, weight loss was independently associated with higher risk of all-cause mortality, and weight gain was not associated with better survival.

**Clinical Trial Registration:** https://clinicaltrials.gov, Identifier: NCT00094302.

## Introduction

Prior studies (1–3) of patients with established heart failure (HF) demonstrated more favorable prognosis in patients with obesity vs. normal weight. The “obesity paradox” led to further investigations on weight change and mortality in patients with chronic HF. Both the American College of Cardiology/American Heart Association guideline (4), and the European Society of Cardiology guideline (5) in HF have not provide conclusive recommendations about weight control. Several informative studies (6–10) that mainly focus on HF with reduced ejection fraction (HFrEF) have shown that both weight loss and weight gain were associated with poor prognosis. However, robust evidence regarding the relation of weight change and long-term prognosis in heart failure with preserved ejection fraction (HFpEF) is missing, despite HFpEF accounts for over half of the overall HF burden all over the world (11–13). Moreover, prior reviews (14–16) raised the differences in baseline characteristics of patients, including gender and prevalence of comorbidities that may account for the “obesity paradox.” Whether patients' characteristic-related differences existed on weight change and HF prognosis remains unknown.

The TOPCAT (Treatment of Preserved Cardiac Function Heart Failure with an Aldosterone Antagonist) was a large international trial among patients with HFpEF, where the effect of the spironolactone was compared with placebo for mortality and morbidity. The main aim of this analysis was to assess the effect of weight loss or gain over a 1-year follow-up period on subsequent mortality in patients with HFpEF enrolled in the Americas in TOPCAT, with further exploration of the interaction between weight change and patients' characteristics and spironolactone treatment.

## Methods

### TOPCAT Study Design and Objectives

The design of the TOPCAT trial has been described in detail previously (17). Briefly, TOPCAT was a multicenter, international, randomized, double blind, placebo-controlled trial of spironolactone in adults with HFpEF recruited from over 270 clinical sites. The trial was funded by the National Heart, Lung, and Blood Institute as a contract with the Brigham and Women's Hospital (Clinical Coordinating Center) and the New England Research Institute (Data Coordinating Center). Enrollment began in August 2006 and ended in January 2012, and the primary results of the trial were published in April 2014 (18). The primary aim was to determine whether treatment with spironolactone, compared with placebo, can produce a clinically meaningful reduction in the composite outcome of cardiovascular mortality, aborted cardiac arrest, or HF hospitalization in adults with symptomatic HF and documented LVEF ≥45%. All study participants provided written informed consent.

Data on vital signs, including body weight and height, were collected at baseline. Patients were followed at 1, 2, 4, 8, 12, and 18 months, and every year thereafter, at which times data on vital signs, including body weight, were collected. Patients were followed for a median of 3.5 years (18).

For the present study, we excluded (i) patients from Russia and Georgia (*n* = 1,678), given the significant regional differences previously described (19), (ii) missing body weight or body mass index (BMI) <18.5 kg/m^2^ at baseline (*n* = 16), (iii) missing body weight at both 1-year follow-up and the follow-up close to it (8 and 18 months) (*n* = 183), and (iv) died before 1-year follow-up (*n* = 12). Death from all causes was the main outcome.

### Definition of Weight Change and Obesity

Weight change was defined as the change in body weight from the baseline measurement to the end of the first year of follow-up. For 118 participants (7.8%) missing body weight at 1-year follow-up, we impute with measures at 8 or at 18 months if it missing at 8 months. A positive value means increased weight, and a negative value means decreased weight. Patients were classified according to weight change into three strata as follows: weight loss (weight witnessed a decrease of ≥5%), weight stability (weight change <5%), and weight gain (weight witnessed an increase of ≥5%). BMI was analyzed according to weight and height at baseline using the formula weight (kg)/(height in m)^2^. Obesity was defined as BMI ≥ 30 kg/m^2^ based on the criteria defined by the World Health Organization (WHO Technical Report Series, no 854, Geneva, 1999). In present analysis, non-obesity was defined as BMI 18.5 to <30 kg/m^2^.

### Statistical Analysis

Categorical variables were described by frequencies with percentages, and continuous variables were described by median with interquartile ranges (IQR) or mean with standard deviation (SD). Demographic and clinical characteristics were compared among weight change groups using the Kruskal–Wallis test for continuous variables and chi-squared tests for categorical variables.

Cox proportional hazards models were used to estimate hazard ratios (HRs) and 95% confidence intervals for mortality, starting from the first year follow-up, associated with weight loss and weight gain using weight stability as reference. Multivariable models adjusted for age, gender, race, smoking status, New York Heart Association (NYHA) class, estimated glomerular filtration rate (eGFR), heart rate, systolic blood pressure (SBP), ejection fraction, diabetes status, atrial fibrillation, peripheral arterial disease, previous hospitalization for HF, prior myocardial infarction, stroke, chronic obstructive pulmonary disease (COPD), baseline BMI, presence of edema, and assignment to spironolactone vs. placebo, using stepwise selection. Covariates were chosen based upon a combination of clinical relevance and previous prognostic implication in the TOPCAT. In addition, we did the Cox regression multivariable analyses using standardized weight change as continuous variable (with 1 SD decrease). To assess for possible non-linearity, we fitted restricted cubic spline models with five knots at the 5, 25, 50, 75, and 95th percentiles of standardized weight change.

Subgroups analyses were conducted to explore interactions on weight change and mortality. Cox regression multivariable analyses using weight change as both categorical and continuous variable were repeated after stratifying patients into different subgroups as follows: obesity or non-obesity, with or without diabetes mellitus, women or men, and allocated to spironolactone or placebo.

All statistical analyses were conducted using SAS statistical software version 9.4 (SAS Institute Inc.), and the forest plot was made using Excel version 15.23 (Microsoft Institute Inc.). All comparisons were two sided, and *P* < 0.05 was considered statistically significant.

## Results

### Baseline Characteristics

Among all study populations, 1,515 participants met the inclusion criteria for the present analysis. The mean (SD) age was 71.5 (9.6) years; 49.4% were women, and 79.1% were White. The median weight change was −0.45 kg (IQR −3.63 to 2.76, range −50.3 to 27.2) during the first year of follow-up. Among all patients, 19.3% experienced weight loss, and 15.9% experienced weight gain. Table 1 lists the baseline characteristics of the study population, stratified by weight change groups. Patients in the weight loss group were more often current smoker, more often had history of stroke and COPD, and less often had history of hypertension, and patients in the weight gain group were younger age, more commonly had diabetes mellitus and previous hospitalization for HF, and less commonly had history of stroke.

**Table 1:** Baseline demographic and clinical characteristics by weight change groups.

	**All (1,515)**	**Weight loss (*n* = 293)**	**Weight stability (*n* = 981)**	**Weight gain (*n* = 241)**	***p***
Weight change, mean (SD), (kg)	−0.5 (6.43)	−9.1 (5.8)	−0.2 (2.6)	8.6 (4.4)	<0.001
**Demographic**					
Age, median (IQR), year	72 (64–79)	73 (63–79)	73 (65–80)	68 (62–76)	<0.001
Women, *n* (%)	749 (49.4)	162 (55.3)	471 (48.0)	116 (48.1)	0.083
Race, *n* (%)					0.186
White	1,199 (79.1)	223 (76.1)	790 (80.5)	186 (77.2)	
Black	247 (16.3)	56 (19.1)	144 (14.7)	47 (19.5)	
**Clinical**					
Randomization to spironolactone, *n* (%)	771 (50.9)	151 (51.5)	502 (51.2)	118 (49.0)	0.803
Current smoker, *n* (%)	89 (5.9)	25 (8.5)	48 (4.9)	16 (6.6)	0.040
**Medical history**, ***n*** **(%)**					
Previous hospitalization for CHF	873(57.6)	166 (56.7)	538 (54.8)	169 (70.1)	<0.001
Previous myocardial infarction	323 (21.3)	59 (20.1)	217 (22.1)	47 (19.5)	0.587
Stroke	141 (9.3)	36 (12.3)	91 (9.3)	14 (5.8)	0.036
COPD	239 (15.8)	58 (19.8)	139 (14.2)	42 (17.4)	0.048
Hypertension	1,360 (89.8)	250 (85.3)	891 (90.8)	219 (90.9)	0.030
Peripheral arterial disease	182 (12.0)	36 (12.3)	123 (12.5)	23 (9.5)	0.433
Atrial fibrillation	657 (43.4)	121 (41.3)	437 (44.5)	99 (41.1)	0.470
Diabetes mellitus	667 (44.0)	120 (41.0)	420 (42.8)	127 (52.7)	0.011
**Physical examination**					
NYHA class III/IV, *n* (%)	515 (34.0)	117(39.9)	316 (32.2)	82 (34.0)	0.051
Presence of edema, *n* (%)	1,077 (71.1)	213 (72.7)	707 (72.1)	157 (65.1)	0.232
Heart rate, median (IQR), (bpm)	68 (60–76)	68 (62–76)	68 (60–75)	68 (61–76)	0.532
SBP, median (IQR), (mmHg)	129 (118–138)	126 (116–138)	130 (118–138)	128 (118–138)	0.122
Body mass index, median (IQR), (kg/m^2^)	32.8 (28.1–38.4)	33.1 (27.9–39.4)	32.7 (28.2–37.9)	32.9 (28.0–39.1)	0.525
**Laboratory and imaging testing, median (IQR), %**					
Ejection fraction	59 (53–65)	57 (53–60)	60 (53–65)	59 (51–65)	0.094
eGFR	61.6 (49.6–76.5)	63.0 (51.1–78.1)	60.8 (48.9–75.1)	63.5 (49.8–79.4)	0.119
**Medication**					
Diuretics	1,343 (88.6)	255 (87.0)	876 (89.3)	212 (88.0)	0.550

*COPD, chronic obstructive pulmonary disease; CHF, chronic heart failure; NYHA, New York Heart Association; SBP, systolic blood pressure; eGFR, estimated glomerular filtration rate*.

### Association of Weight Change and All-Cause Mortality

**Figure 1:**
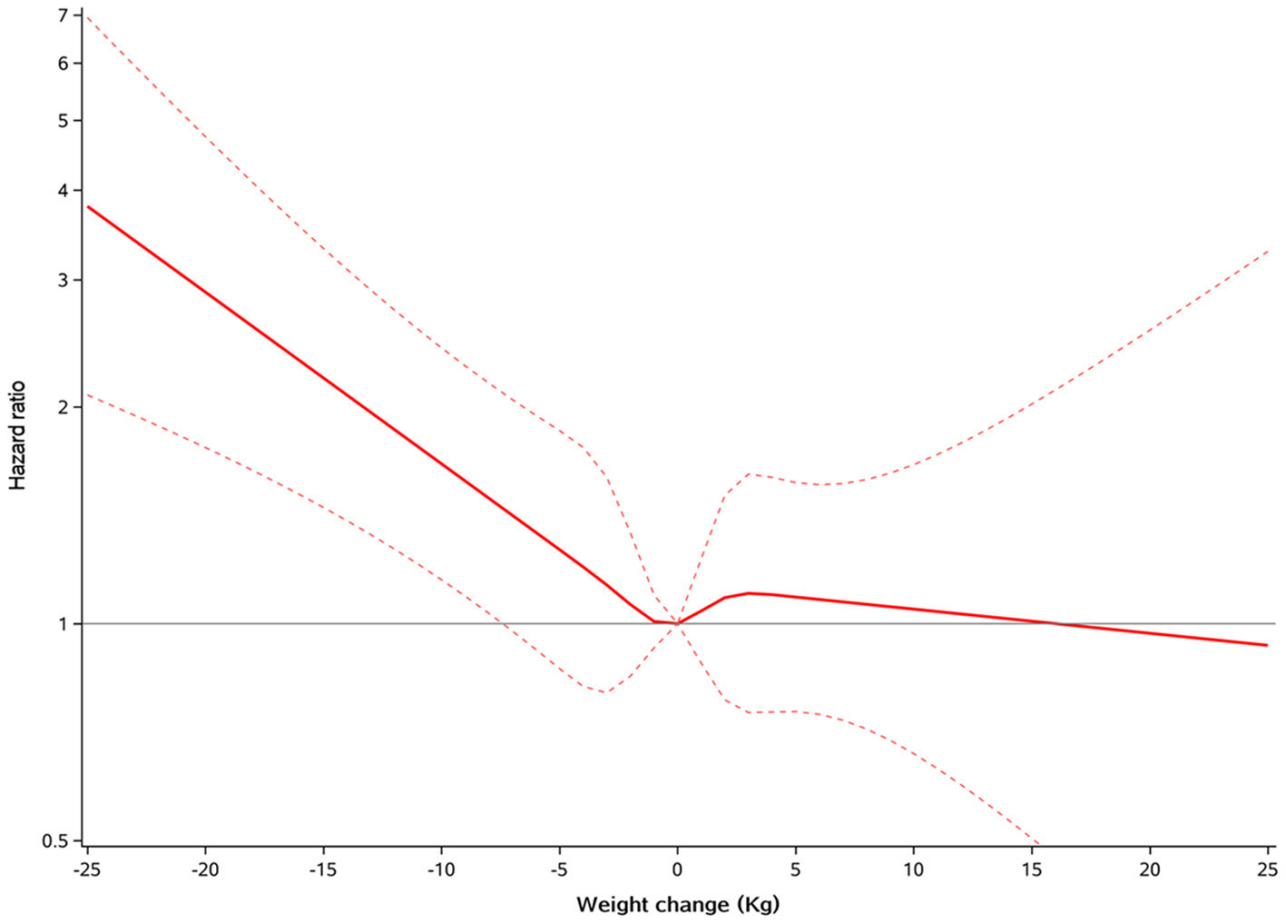
Restricted cubic spline plots for all-cause mortality by weight change. A positive value means increased weight, and a negative value means decreased weight.

During a mean subsequent follow-up of 2.5 years after the first year, all-cause mortality occurred in 65 (22.2%), 175 (17.8%), and 36 (14.9%) patients with weight loss, weight stability, and weight gain, respectively. In the multivariable model adjusted for age, gender, race, smoking status, NYHA class, eGFR, heart rate, SBP, ejection fraction, diabetes status, atrial fibrillation, peripheral arterial disease, previous hospitalization for HF, prior myocardial infarction, stroke, COPD, baseline BMI, presence of edema and assignment to spironolactone, weight loss was associated with a higher risk of mortality (HR 1.42, 95% CI 1.06–1.89, *P* = 0.002), and weight gain had similar risk of mortality (HR 0.98, 95% CI 0.68–1.42, *P* = 0.932), compared with weight stability (Table 2). Findings from restricted cubic spline analysis demonstrate that there was a linear relationship between weight change as a continuous variable and all-cause mortality (*P* = 0.194 for overall relationship) (Figure 1). Similar linear relationship was found between relative changes in body weight and mortality (Supplementary Figure 1). One SD decrease in weight was associated with 21% higher risk of mortality (HR 1.21, 95% CI 1.08–1.36, *P* = 0.001) (Supplementary Table 1).

**Table 2:** Multivariable Cox regression analysis for all-cause mortality.

**Covariates**	**HR**	**95% CI**	***p***
Weight loss^*^	1.42	1.06–1.89	0.018
Weight gain^*^	0.98	0.68–1.42	0.932
SBP	0.99	0.98–1.00	0.008
Age	1.04	1.03–1.06	<0.001
Women	0.63	0.49–0.81	<0.001
Black race^#^	1.90	1.15–3.12	0.012
Other race^#^	0.90	0.60–1.35	0.624
Previous hospitalization for CHF	1.37	1.06–1.76	0.015
Diabetes mellitus	1.43	1.11–1.85	0.006
eGFR	0.99	0.98–1.00	0.007

* *Using weight stability as reference*.

# *Using white race as reference*.

*SBP, systolic blood pressure; eGFR, estimated glomerular filtration rate*.

### Subgroup Analysis

**Figure 2:**
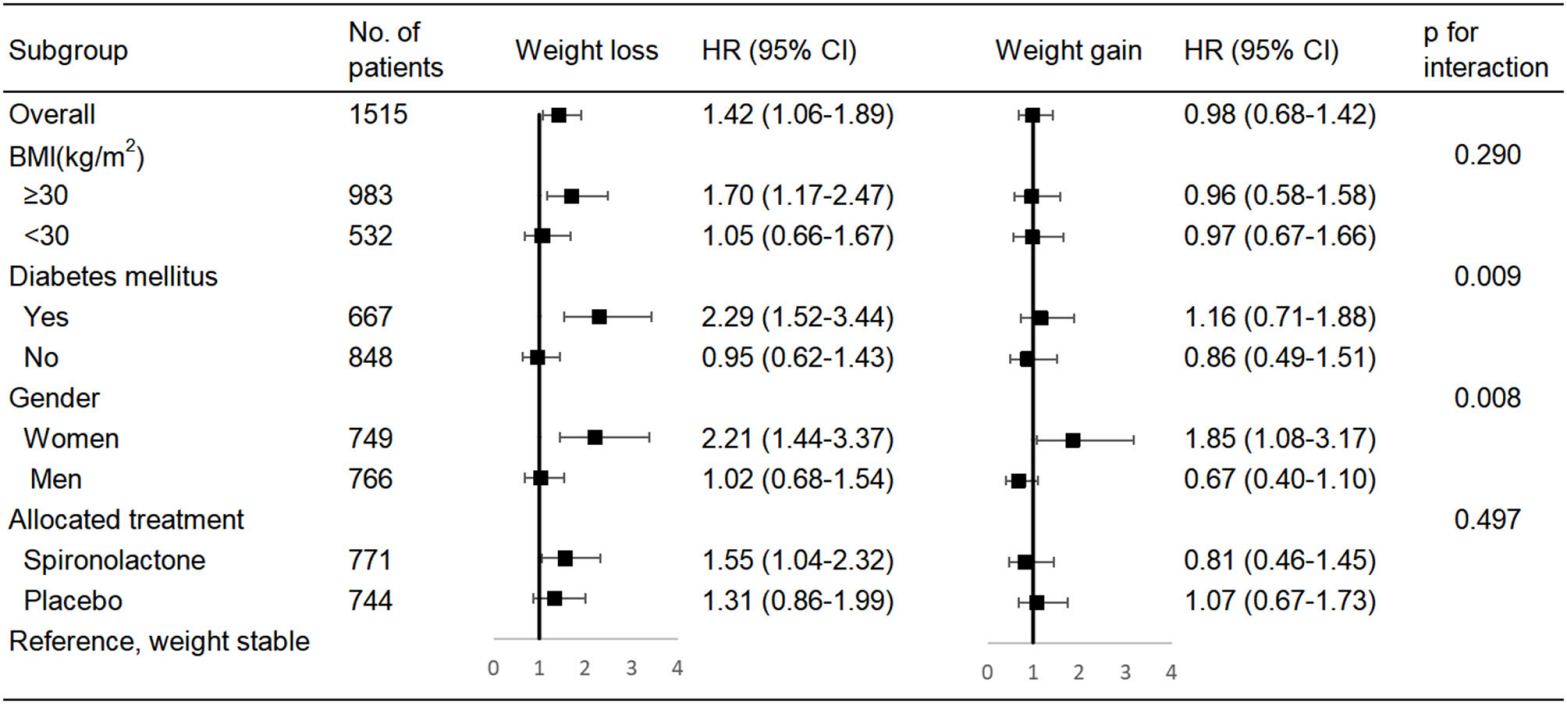
Multivariable Cox regression analysis for various subgroups. BMI, body mass index.

Figure 2 shows the association between weight change groups and all-cause mortality for several patient subgroups. We explored whether the link between weight change and mortality risk was different for patients with and without obesity: no such interaction was found. However, among HF patients with obesity, weight loss was associated with higher risk of mortality than that observed in patients without obesity. The impact of weight loss on mortality was related to diabetes mellitus (interaction *p* = 0.009). Weight loss was significantly associated with remarkable higher mortality among patients with diabetes mellitus (adjusted HR 2.29, 95% CI, 1.52–3.44, *P* < 0.001), but was non-significant among patients without diabetes mellitus (adjusted HR 0.95, 95% CI, 0.62–1.43, *P* = 0.793). A similar interaction was found using weight change continuous variable (interaction *p* = 0.02) (Supplementary Figure 2). The impact of weight loss on mortality appeared more pronounced in women (interaction *p* = 0.008), but no such interaction was found when using weight change as continuous variable. The link between weight loss and mortality risk was similar in patients on spironolactone and on placebo.

## Discussion

We have found that both weight loss and weight gain were common in patients with HFpEF. Weight loss was associated with increased mortality risk from all causes, and weight gain was not associated with lower mortality risk. In addition, the impact of weight loss on mortality may be interacted by diabetes status and gender. Findings from the current study extended previous evidence to a less known population, HFpEF, and raised novel interaction in a broad spectrum of subgroup analysis.

Unintentional weight loss was witnessed in 14–21% of patients with HF in prior studies (6–8, 20), with results quite similar to ours. These studies have provided important information on association of weight loss with outcomes in HF. Anker et al. (21) first demonstrated that weight loss of at least 7.5% during at least 6 months in HF was an independent risk factor for poor prognosis in a small-sample and single-center study. *Post-hoc* analysis of the SOLVD and V-HeFT II trials (10) identified which level of weight loss gave the strongest discrimination and proposed 6% of weight loss to define cachexia in HFrEF. In an analysis of the CHARM study (9), those patients with 5% or greater weight loss in 6 months had over 50% increase in hazard both for mortality compared with those with stable weight. Analysis of patients with HFrEF from the Val-HeFT study (7) found that 5% or greater weight loss in 1 year was independently associated with mortality and adverse cardiovascular outcomes. Zamora et al. (6) also reported that 5% or greater weight loss in 1 year was associated with an increased 89% risk of mortality in patients with ambulatory HFrEF. The present study is the first one focusing on patients with HFpEF, which accounts for over half of the HF population. We demonstrated that weight loss was also an independent prognostic factor in patients with HFpEF, that 5% or greater weight loss in 1 year was associated with an increased 42% risk of subsequent long-term mortality compared with patients with stable weight. More precise estimation achievable with restricted cubic spline demonstrated that 1 SD decrease in weight was associated with 21% higher risk of mortality. Thus, in addition to routine monitoring of body weight that was recommended by HF guidelines, calling for vigilance on apparent weight loss is also suggested throughout long-term HF management.

Although lacking in robust evidence, the potential benefit of intentional weight loss was suggested in several pilot studies in established HF patients with obesity. Weight loss through bariatric surgery and non-surgical approaches has been found to improve LVEF (22, 23), exercise capacity (24), NYHA class (25, 26), and quality of life (27, 28). The controversy effect between intentional and unintentional weight loss suggested different mechanisms during this course. The onset of unintentional weight loss may be a signal of HF progress to imbalance between catabolic and anabolic states, and the subsequent wasting outlook of the patients. A few studies (10, 29, 30) have found hormonal and immune activations such as interleukin-6 and tumor necrosis factor-α in patients with cardiac cachexia. Nevertheless, further research on the underlying mechanisms are still needed. In a previous study (24) on intentional weight loss by caloric restriction or aerobic exercise training, the change in peak Vo2 was positively correlated with the change in percent lean body mass and the change in thigh muscle:intermuscular fat ratio. Another study (31) assessing mortality based on body fat and lean mass, rather than BMI or weight alone, reported that subjects losing body fat, rather than lean mass, have a lower mortality. Thus, improvement in body composition, instead of indiscriminate weight loss, is a promising target in future HF programs.

Unintentional weight gain in established HF was less investigated in previous studies. This study showed that weight gain was almost as frequent as weight loss in patients with HFpEF. The *post-hoc* analysis of the CHARM study (9) found that weight gain was associated with modestly increased short-term mortality risk. Similar results were also reported in the sub-analysis of patients from the GISSI-HF and Val-HeFT studies (7). On the contrary, we demonstrated the neutral role of weight gain on mortality risk compared with weight stability in patients with HFpEF, and results from the restricted cubic spline analysis confirmed this association. Difference in HF population may account for this discrepancy that the majority of patients enrolled in prior studies was HFrEF. Hitherto, there has been no evidence that patients with established HF can benefit from weight gain. We demonstrated that weight gain was not associated with better prognosis even in HF patients without obesity.

Notably, the effect of weight loss on all-cause mortality was remarkable among patients with diabetes mellitus, but was non-significant among patients without diabetes mellitus. One possible explanation is that unintentional weight loss may result from insufficient antidiabetic treatment, and the body subsequently starts burning fat and muscle for energy in patients with diabetes mellitus. Such unintentional weight loss related to progression of disease would be expected to increase mortality (32). We also show that the link of weight loss to mortality may be different between women and men in established HF, whereas this gender difference need to be tested in a larger study. The CHARM study (9) showed that the impact of weight loss on mortality appeared more pronounced in patients not receiving angiotensin-converting-enzyme inhibitors (ACEI) (interaction *P* = 0.01) compared with those receiving ACEI. However, no such interaction was observed for spironolactone in the present analysis.

There are several limitations to our study because participants were from a clinical trial database that had several exclusion criteria that might affect the generalizability. The cutoff of weight change equal to or <5% can be considered arbitrary as were all the definitions used in previous studies (6, 7); however, no definite cutoff exists. We have no further anthropometric measures (muscle or fat mass wasting assessments), and we cannot fully ascertain whether weight change was in part intentional or non-intentional. Although we have adjusted multiple patient characteristics including presence of edema at baseline, a higher prevalence of relevant risk factors, such as COPD in the weight-loss group, and the average younger age in the weight-gain group may have played a role in the incidence of death, and bias due to unmeasured confounders are possible. Due to the limitation of the sample size, we did not analyze the specific cause of death.

In conclusion, this study shows that weight loss is an independent factor of poor prognosis in HFpEF with normal to overweight, especially in patients with diabetes, though this interaction needs further investigation. Weight gain was not associated with better prognosis, either. Indiscriminate advice to lose or gain weight in HFpEF might not be indicated, and the underlying mechanism of weight change on mortality merits further research.

## Data Availability Statement

The datasets presented in this study can be found in online repositories. The names of the repository/repositories and accession number(s) can be found in the article/Supplementary Material.

## Ethics Statement

The studies involving human participants were reviewed and approved by the ethics committee of the First Affiliated Hospital of Sun Yat-sen University. The patients/participants provided their written informed consent to participate in this study. Ethical review and approval was received for the original clinical trial.

## Author Contributions

BD, PH, and ZG design the research. CL, WL, YW, XH, and WZ analyse the data. PH, JZ, YD, and YY write the article. All authors contributed to the article and approved the submitted version.

## Conflict of Interest

The authors declare that the research was conducted in the absence of any commercial or financial relationships that could be construed as a potential conflict of interest.
