# Supplementary material for: Weight Change and Mortality Risk in Heart Failure With Preserved Ejection Fraction
Source: Front Cardiovasc Med. 2021 Jun 4;8:681726. doi: 10.3389/fcvm.2021.681726 (PMC8213199; doi:10.3389/fcvm.2021.681726)
Supplement: Supplementary file 1 [file Data_Sheet_1.docx]

Supplemental material

Supplementary Table 1. Multivariable cox regression analysis for all-cause mortality, using weight change as continuous variable.

| Covariates | HR | 95% CI | p |
| --- | --- | --- | --- |
| Weight loss* | 1.21 | 1.08-1.36 | 0.001 |
| SBP | 0.99 | 0.98-1.00 | 0.010 |
| Age | 1.04 | 1.03-1.06 | <.001 |
| Women | 0.61 | 0.47-0.77 | <.001 |
| Previous hospitalization for CHF | 1.38 | 1.07-1.77 | 0.012 |
| Diabetes mellitus | 1.42 | 1.10-1.84 | 0.007 |
| eGFR | 0.99 | 0.98-1.00 | 0.007 |

* Per 1 SD decrease in weight.

SBP, systolic blood pressure; eGFR, estimated glomerular filtration rate.

Supplementary Figure 1. Restricted cubic spline plots for all-cause mortality by relative weight change.


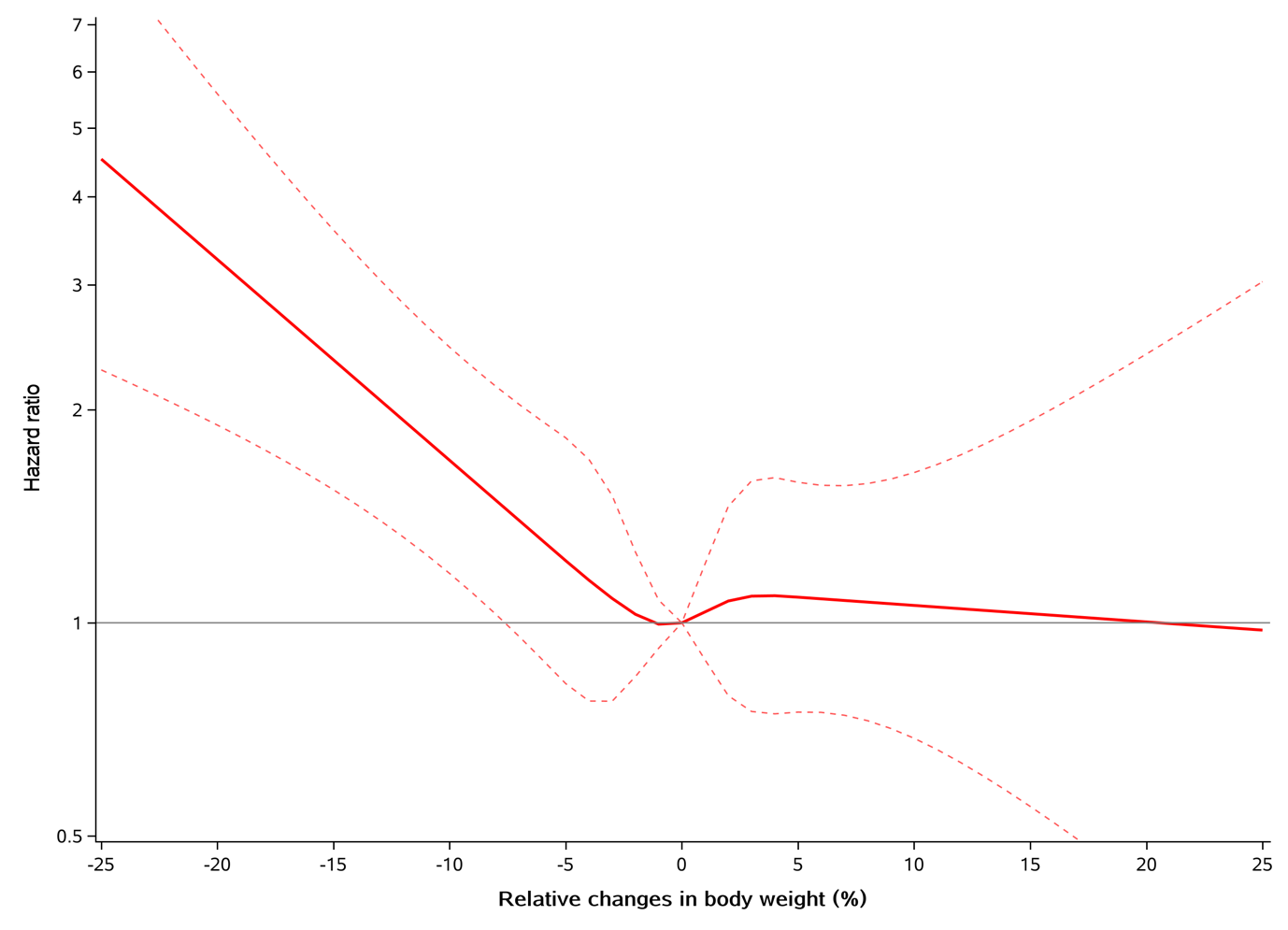


Supplementary Figure 2. Multivariable cox regression analysis for per 1 SD decrease in weight on all-cause mortality, for various subgroups.


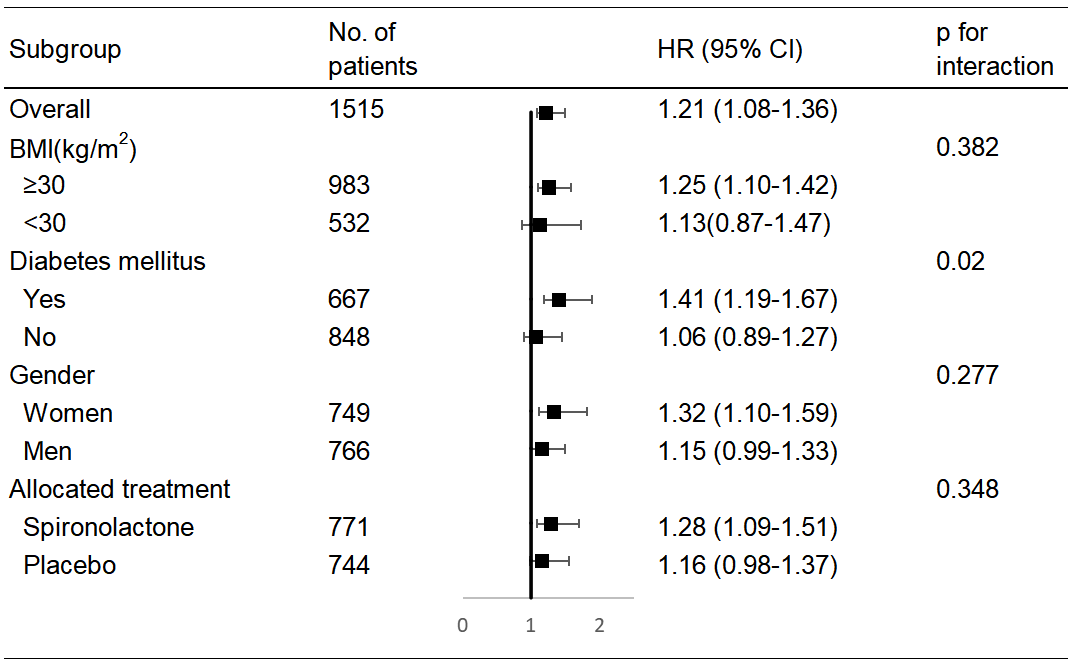


BMI, body mass index
